# Special Issue “Fibre Optic Sensors for Structural and Geotechnical Monitoring”

**DOI:** 10.3390/s20082415

**Published:** 2020-04-24

**Authors:** Michele Arturo Caponero

**Affiliations:** Photonics Micro and Nanostructures Laboratory, ENEA Research Centre of Frascati, Via Enrico Fermi 45, Frascati, 00044 Rome, Italy; michele.caponero@enea.it

## Abstract

In this editorial on the special issue “Fibre Optic Sensors for Structural and Geotechnical Monitoring” a review of the contribution papers selected for publication is given. Each paper is briefly summarized, presenting its objective and methods, then a comment is given about the relevance of the work with respect to the advance and the spreading of the fibre optic technology for monitoring applications.

## 1. Introduction

The use of sensors based on fibre optic technology allows a broad range of applications in the fields of structural and geotechnical monitoring, which can effectively improve maintenance of infrastructures and safety of communities. Thanks to its valuable features, such as distributed monitoring, easy and endurance of cabling, long term stability, reliable response in both static and dynamic regime, fibre optic technology has already provided innovative and efficient solutions to quite difficult monitoring problems. The worldwide increasing attention to infrastructures and communities with resilience capabilities against natural disasters has opened up new and challenging prospective applications for the use of fibre optic technology for structural and geotechnical monitoring. 

This Special Issue collects contributions in the development and application of monitoring solutions based on fibre optic technology for structural and geotechnical engineering works and issues. In the following, the content of the contributions is reviewed, providing a brief introduction to the work presented in the paper and commenting the relevance of the work with respect to the advance and the spreading of the fibre optic technology for monitoring applications. All contributions, following the recommendation in the invitation to this Special Issue, provide a comprehensive discussion and report a rich bibliography on the current trends and the issues relative to the work presented in the paper.

## 2. Contributions

Paper [1] reports the monitoring activity successfully tested in a marble quarry, by Brillouin sensing combined with drone photogrammetry and geotechnical survey. The monitoring activity is intended to both raise the safety at work and assist the extraction planning. Beyond the specific application, the paper confirms the effective capability of fibre optic distributed sensing techniques to monitor complex geomorphological sites. Moreover, the paper proposes and demonstrates an efficient procedure to integrate fibre optic distributed sensors in existing monitoring systems based on an array of spatially localized geotechnical sensors (extensometers, crackmeters, inclinometers, topographic markers, etc.). That is a procedure of relevant interest, as it can facilitate the spreading of fibre optic distributed sensing, which can thus be proposed as an efficient tool to upgrade monitoring installations based on traditional technologies.

Paper [2] reports the successful demonstration, on a small mockup, of the use of fibre optic distributed sensing to characterize plate foundation support. As the execution of distributed strain measurements with very high-resolution is critical to the demonstration on the small mockup, a suitable technique is adopted, which is based on a proprietary implementation of the optical frequency domain reflectometry. In the perspective of future demonstration on a true size mockup and with the envision of engineering application on real works, since very high-resolution will not be necessary, the authors preview the use of standard fibre optic distributed sensing techniques. Beyond the specific application, the paper confirms the high versatility of the fibre optic distributed sensing technologies, that prove themselves as a major candidate for the development of self-monitoring extended engineering structures.

Paper [3] reports the use of fibre optic distributed monitoring to investigate properties of geomaterial samples according to the laboratory procedure named direct simple shear test. A new procedure is proposed to evaluate some component of the stresses in the sample, taking advantage of the very high-resolution measurements that can be done by use of a proprietary implementation of the optical frequency domain reflectometry. The paper presents the production of an upgraded sample holder for direct simple shear test, the execution of the test, and a discussion of the results. The sample holder is made by 3D printing, with grooves in which the optical fibre distributed sensor is installed. The paper finely demonstrates the use of high-resolution fibre optic distributed sensing for the direct simple shear test. The paper, with a new application of a fibre optic sensing technique to laboratory tests, assesses the broad range of potential applications of the fibre optic sensors in the geothechnical field, so far mostly proved for large-scale in-field applications. 

Paper [4] reports a novel method of data processing for distributed acoustic sensing by phase-sensitive optical time domain reflectometry. The method, based on spatial kurtosis, is intended to improve the signal detection in noisy systems and in presence of environment perturbations (high signal to noise ratio). The method is validated in laboratory and outdoor experimental tests, simulating structural cracking and malicious digging. Results provide a valid perspective of the broadening of use of distributed acoustic sensing for applications in structural health monitoring and intrusion surveillance. In fact, the method for basic (no proprietary) optoelectronic hardware meets requirements for real-time data analysis and does not lower the spatial resolution provided by other assessed signal processing methods. 

Paper [5] reports an application of distributed temperature sensing to monitor the final construction phase and commissioning of wells serving a CO_2_ geological storage. In the paper, offline data analysis is done adopting different temperature calibration methods and results are compared. Beyond the specific application, the paper addresses practical procedures to face the impossibility to properly perform calibration of cables before installation, which is an often-recurring situation when working in a real construction yard. The paper provides a relevant example of an application in which the same fibre optic distributed monitoring installation can be first used to monitor first the correct production of the infrastructure, and can be later used to monitor it along its working life. Moreover, the installed sensing line is shown to have fibres for distributed acoustic sensing too, which makes the wells a relevant potential demonstration site for multifunctional monitoring by fibre optic distributed monitoring. 

Paper [6] reports an application of structural health monitoring of a railway tunnel. Monitoring is done by strain gauges and pressure cells based on the fibre Bragg grating technology. Monitoring is focused on the interaction soil-tunnel, the tunnel being built in open space and later buried. The work done shows an impressive engineering effort, with the adoption of rugged solutions to protect the installation from the severe working yard conditions. Moreover, a valuable system for remote control and automatic data collection is at service of the installation. Beyond the specific application, the paper shows the high maturity of the technological solutions available to develop monitoring systems based on the the fibre Bragg grating sensors which offer an unrivalled versatility in the production of customised and multifunctional chain of sensors.

Paper [7] reports use of fibre Bragg grating sensors in a real size test on prefabricated concrete hollow piles. The test is intended to characterize the performance of the piles during the installation and the subsequent loading test. Chains of sensors were were installed on the external surface of the piles along diametrically opposite longitudinal grooves. Piles were installed in clay subsoil by a clamping-and-jacking machine, with a step-by-step pushing action. The loading test was done 17 days later, with an hydraulic jack inserted between the pile upper end and loading platform loaded with concrete blocks. Results provide an effective characterization of the performance of the piles. Beyond the specific application, the work points out the potential of the fibre Bragg sensor technology in solving problems related to the characterization and monitoring of the civil and geotechnical engineering infrastructures. In fact, the technology offers both fast response to dynamic or transitory events and long term stability to static loads, which are paramount features whenever measurements shall be done before and after the production, with some curing/settlement time to wait for.

Paper [8] presents a displacement sensor developed for structural health monitoring in the civil engineering field, with specific potential applications to monitor spring dampers for floating slab installation. The displacement sensor design is based on a sliding block that affects the deflection of a cantilever; the cantilever deflection is monitored by two fibre Bragg grating sensors. The paper presents a prototype sensor dimensioned and produced for an intended application on floating slabs of a subway line in Beijing. The performance of the prototype is investigated for sensitivity in the full displacement range, repeatability, temperature compensation capability and creep. The paper shows the high versatility and potentiality of fibre Bragg grating sensors in being used to equip machinery transducers for mechanical measurements. The use of such sensors for transducers devoted to the structural health monitoring of civil engineering infrastructures can effectively benefit of their peculiar features, as for instance the data taking in wavelength division multiplexing which can greatly lower the cost of installations with many sensors to be deployed with long routing.

Paper [9] investigates the structural health capabilities of fibre Bragg grating sensors embedded in components made by ultrasonic additive manufacturing. Sensors are embedded during the manufacturing process of standard specimens for laboratory crack tests. During the tests, the effectiveness of the sensors in detecting the crack and its evolution is characterised. Sensors are also thermally tested to investigate the upper temperature operational value in the embedded condition. The paper contribute to assess the versatility of the fibre Bragg grating sensors as embeddable sensors in components made by special metallurgic process. Thus, being already assessed since long the possibility of their embedding in carbon and glass composite materials, fibre Bragg grating sensors demonstrate their unrivalled potential to provide prognostic capabilities to high technology mechanical components.

Paper [10] reports the use of fibre Bragg grating sensors to characterize an innovative composite component of aircraft landing gear. Several sensors are used to monitor strain while performing mechanical tests to simulate in laboratory the expected operating stress condition. Sensors are glued on the surface of the component, and preliminary tests are done to select the best adhesive for the specific application. Preliminary tests also confirm that no spectral distortion of the signal of the sensors occurs in the full range of the load test, which guarantee both the correct working condition of the sensors the correct decoding of their spectroscopic signal. Beyond the specific application, the paper confirms the potential of fibre Bragg grating sensors to be used for fine mechanical measurements, with easy production of minimally invasive and easy cabling multifunctional chain of sensors. Moreover, the spectral signature of the sensors provides a reliable feature to control their correct functioning.

Paper [11] reports a demonstration of distributed dynamic sensing by use of ultra-weak fibre Bragg grating technology. The test site is a subway tunnel in regular service, the demonstration is for intrusion alarm and structural heath monitoring. Distribute sensing is tested for a length of 5 km, with spatial resolution of 5 m. Sensing is done by optical time domain reflectometry to evaluate vibration position and by phase demodulation to evaluate frequency and amplitude of the vibration. Beyond the specific application, the paper confirms the availability of emerging and promising techniques for distributed acoustic sensing that can go beyond the limits of the traditional ones and can manage the demanding specifications often required for retrofitting interventions on existing large civil infrastructure, as for instance high sensitivity, real-time response, operatibility with high signal-to-noise ratio.

Paper [12] proposes and tests in the laboratory a method for corrosion detection and evaluation of concrete rebars. The method is applied by use of an array of long gage sensors, each sensor being made by a fibre Bragg grating installed in a tubular housing whose length defines the sensor length gauge. The array of sensors is used to perform strain modal identification, in turn used to perform corrosion detection and evaluation, working out both location and quantification. The proposed method is tested and validated on a reinforced concrete beam subjected to controlled accelerated corrosion procedure. The paper is a relevant example of innovation procedure for structural health monitoring of reinforced concrete infrastructures, whose implementation is facilitated by the unrivalled property of the fibre Bragg grating technology in providing both dynamic and stable static strain monitoring.

Paper [13] proposes a strain measurement procedure based on the bending loss variation of an optical fibre specially arranged on the component to be monitored. The special arrangement consists of a short length of the optical fibre stretched and twisted around a few small cylinders. Sensing is encoded in the intensity of the light transmitted trough the fibre. The cylinders are attached to the component to be monitored, with the correct pattern according to the strain measurement to be done. As the pattern of the cylinders changes according to the deformation experienced by the component, the bending of the fibre changes accordingly and in turn a variation of the bending loss occurs. The paper presents the results obtained with composite specimens subject to tensile and bending tests. The paper is an interesting example of the use of basic properties of the optical fibre and simple setup to perform strain measurements with the possibility to easily adjust both sensitivity and gauge length.

Paper [14] proposes a deflection sensor based on the bending loss of machined plastic optical fibre. The sensor is intended for application in structural health monitoring of civil and geotechnical infrastructures. In particular, its use is proposed as a permanent monitoring device to be used with scheduled surveys by topographic techniques. The sensor is in the form of a bar with four plastic fibres running longitudinally on its surface, equally spaced along its circumference. The sensing element is a short segment of the plastic fibre, machined to have saw-teeth-shaped groves on its cladding in order to maximize bending losses. The four sensing elements monitor the bending of the bar, which has to be stuck on the structure to be monitored for deflection. The paper is an interesting example of production of a modular and cost-effective monitoring tool by use of plastic fibres and exploitation of the basic properties of guided light transmission.

Paper [15] proposes and studies a bending sensor intended for structural health monitoring of underwater civil engineering structures. The sensor is based on the use of a plastic fibre and specific pulsed light-emitting and power-measuring circuitry. Along the plastic fibre, a short sensing segment is produced by making a machined cut which acts as a light leakage zone. Leakage depends on the local bending of the fibre, thus power loss encodes the measurement of the local bending. The paper presents and verifies a model of the bending loss mechanism having parameters which related to the geometry of the leakage zone. Laboratory tests validate a prototype of the proposed sensor and provide its experimental characterisation. The paper is a relevant example of the application oriented development of a sensor based on a basic property, namely the bending loss, whose metrological features are upgraded with respect of the state of the art thanks to simple but effective encoding/decoding signal solutions.
